# Reactivation of Desensitized Formyl Peptide Receptors by Platelet Activating Factor: A Novel Receptor Cross Talk Mechanism Regulating Neutrophil Superoxide Anion Production

**DOI:** 10.1371/journal.pone.0060169

**Published:** 2013-03-28

**Authors:** Huamei Forsman, Karin Önnheim, Emil Andréasson, Karin Christenson, Anna Karlsson, Johan Bylund, Claes Dahlgren

**Affiliations:** Department of Rheumatology and Inflammation Research, University of Gothenburg, Göteborg, Sweden; Medical School of Hannover, United States of America

## Abstract

Neutrophils express different chemoattractant receptors of importance for guiding the cells from the blood stream to sites of inflammation. These receptors communicate with one another, a cross talk manifested as hierarchical, heterologous receptor desensitization. We describe a new receptor cross talk mechanism, by which desensitized formyl peptide receptors (FPR_des_) can be reactivated. FPR desensitization is induced through binding of specific FPR agonists and is reached after a short period of active signaling. The mechanism that transfers the receptor to a non-signaling desensitized state is not known, and a signaling pathway has so far not been described, that transfers FPR_des_ back to an active signaling state. The reactivation signal was generated by PAF stimulation of its receptor (PAFR) and the cross talk was uni-directional. LatrunculinA, an inhibitor of actin polymerization, induced a similar reactivation of FPR_des_ as PAF while the phosphatase inhibitor CalyculinA inhibited reactivation, suggesting a role for the actin cytoskeleton in receptor desensitization and reactivation. The activated PAFR could, however, reactivate FPR_des_ also when the cytoskeleton was disrupted prior to activation. The receptor cross talk model presented prophesies that the contact on the inner leaflet of the plasma membrane that blocks signaling between the G-protein and the FPR is not a point of no return; the receptor cross-talk from the PAFRs to the FPR_des_ initiates an actin-independent signaling pathway that turns desensitized receptors back to a signaling state. This represents a novel mechanism for amplification of neutrophil production of reactive oxygen species.

## Introduction

The seven transmembrane receptor (7TMR) family of G protein-coupled receptors (GPCRs) is a large and diverse group of cell surface receptors important for many cellular activities, e.g., proliferation, differentiation, growth, and death. The involvement of 7TMRs in the regulation of inflammatory cells, e.g., mediating chemotaxis, is well established [1]. Most cellular responses triggered by these receptors are induced by a generally accepted 7TMR-signaling scheme. First, ligand binding stabilizes the occupied 7TMR in an active signaling conformation during which the bound heterotrimeric G-protein dissociates into subunits that regulate the activity of enzymes such as adenylate cyclases, phospholipase C isoforms, kinases, as well as ion channels, resulting in generation of small-molecule second messengers that control cellular functions [2]. Subsequently, signaling is terminated (or switches direction towards endocytic uptake of the receptor-ligand complex) and the occupied receptor becomes refractory to further stimulation with the same agonist, an effect commonly termed homologous desensitization [3], [4]. One mechanism suggested to account for both termination of signaling and receptor desensitization is receptor phosphorylation and binding of arrestin to the cytosolic parts of the agonist-occupied receptor [5], [6]. According to this model, binding of arrestin causes occlusion of the heterotrimeric G-protein [7], [8], [9], [10].

Formyl peptide receptor 1 (FPR1), the prototype chemoattractant 7TMR in neutrophil granulocytes [11], [12], recognizes formylmethionyl-containing “danger” peptides derived from microbes and mitochondria [13], [14]. The 7TMR signaling pathway described above is valid for FPR1, with the exception that, although this receptor binds arrestin [15], this protein does not seem to be the key protein for termination of signaling [16]. Instead, cytoskeletal actin plays a more direct and important role in FPR1 termination/desensitization [17], [18], [19]. Irrespective of desensitization mechanism the resulting non-signaling state of a ligand-occupied 7TMR is thought to be stable and is the starting point for receptor internalization. No signaling pathway has been described that reverses the desensitized receptor into an active signaling state [20].

Neutrophils are equipped with a membrane-bound electron transporting system, the NADPH-oxidase, that upon activation transfers electrons from cytosolic NADPH to molecular oxygen on the other side of the membrane. The resulting superoxide anion release is of prime importance for our innate immune defence, both killing microbes and mediating regulation of inflammatory reactions [21], [22], [23]. The bactericidal activities of neutrophils rely on the ability of the cell’s to recognize different chemoattractants serving as “danger signals” [24]. In addition to FPR1, neutrophils express the closely related FPR2, receptors for complement component C5a and interleukin-8 (IL8), as well as receptors recognizing lipid metabolites such as leukotriene B4 (LTB_4_) and platelet-activating factor (PAF) [25], [26], [27]. Given that multiple chemoattractants recognized by neutrophil 7TMRs are present simultaneously at sites of inflammation, the outcome of a neutrophil response is likely to be regulated by so-called hierarchical receptor cross talk to ensure that cells can migrate directionally also in opposing gradients of chemoattractants [28]. Such cross talk whereby hierarchically strong (end-point) chemoattractants overrule weaker chemoattractants is mediated by heterologous receptor desensitization [28], [29]. This means that ligation and activation of one (hierarchically strong) receptor may desensitize also non-occupied but hierarchically weaker receptors of other ligand specificities. For example, FPR1 ligands desensitize cells not only to FPR1 agonists, but also to the agonists IL8 and LTB_4_, binding to CXCR1/2 and the BLT1, respectively [30], [31], [32], [33], [34], No desensitization is, however, obtained when the agonist order is reversed [28]. The FPR1 is thus of higher hierarchical order than CXCR1/2 and BLT1. It has been suggested that some receptor pairs, for example FPR1 and PAFR, are hierarchically equal since there is no cross desensitization in either direction [35]. Although single receptor-mediated responses in neutrophils have been much studied, receptor cross talk mechanisms leading to desensitization, and as shown in this study, reactivation, are only beginning to be unraveled.

Here a novel receptor cross talk mechanism, by which the PAFR reactivates occupied and desensitized FPRs, is disclosed. The results presented challenge the view that desensitized receptors stay desensitized without the possibility to reconvene its signaling. To explain this receptor cross talk phenomenon leading to FPR reactivation we have added a new actin-independent mechanism to the earlier described model for receptor desensitization through interactions with the actin cytoskeleton.

## Materials and Methods

### Chemicals

The hexapeptide WKYMVM, the formylated peptide fMIFL, and the PIP_2_-binding peptide PBP10 were synthesized and HPLC-purified by TAG Copenhagen A/S (Copenhagen, Denmark). The FPR2 antagonist WRWWWW was from Genscript Corporation (Scotch Plains, NJ, USA). The formylated fMLF, IL8, isoluminol, latrunculinA and, FITC-labeled phalloidin, were obtained from Sigma (Sigma Chemical Co., St. Louis, MO, USA). Cyclosporin H was kindly provided by Novartis Pharma (Basel, Switzerland). The PAF and its analogues mcPAF and lysoPAF were from Avanti Polar Lipids Inc. (Alabama, USA). Peptides were dissolved in DMSO and stored at −70°C until use. Subsequent dilutions of all reagents were made in Krebs-Ringer phosphate buffer (KRG, pH 7.3; 120 mM NaCl, 5 mM KCl, 1.7 mM KH_2_PO_4_, 8.3 mM NaH_2_PO_4_ and 10 mM glucose) supplemented with Ca^2+^ (1 mM) and Mg^2+^ (1.5 mM). The PAFR antagonist WEB2086 was from Tocris Bioscience (Bristol, UK). Dextran and Ficoll-Paque was obtained from GE-Healthcare Bio-Science (Uppsala, Sweden). Horseradish peroxidase (HRP) was obtained from Boehringer Mannheim (Germany). CalyculinA was purchased from Nordic Biosite (Sweden). The FURA-2 was from Molecular Probes (Eugene, OR).

### Isolation of Human Neutrophils

Human peripheral blood neutrophils were isolated from buffy coats from healthy blood donors using dextran sedimentation and Ficoll-Paque gradient centrifugation as described [36]. The remaining erythrocytes were disrupted by hypotonic lysis, the neutrophils were washed twice, resuspended in KRG, and stored on melting ice until use. This isolation procedure permits cells to be purified with minimal granule mobilization.

### Neutrophil NADPH-oxidase Activity

The NADPH-oxidase activity was determined using isoluminol-enhanced chemiluminescence (CL) [37], [38]. The CL activity was measured in a six-channel Biolumat LB 9505 (Berthold Co., Wildbad, Germany), using disposable 4-ml polypropylene tubes with a 900 µl reaction mixture containing 10^5^ cells, isoluminol (2×10^−5^ M) and HRP (2U). The tubes were equilibrated in the Biolumat for 5 min at 37°C, after which the stimulus (100 µl) was added and the light emission was recorded continuously. Receptor desensitized cells are defined as naïve (non-desensitized) cells that had first been stimulated with receptor-specific agonist and returned to baseline after the resulting release of superoxide. These cells were then stimulated a second time. When experiments were performed with antagonists, the antagonists were added to the CL reaction mixture 1 min before the second stimulation. Control cells received no treatment but were incubated at the same basal condition as stimulated cells.

### Calcium Mobilization

Neutrophils at a density of 1–3×10^6^ cells/ml were washed with Ca^2+^-free KRG and centrifuged at 220×*g*. The cell pellets were resuspended at a density of 2×10^7^ cells/ml in KRG containing 0.1% BSA, and loaded with 2 µM FURA 2-AM for 30 minutes at room temperature. The cells were then diluted to twice the original volume with RPMI 1640 culture medium without phenol red (PAA Laboratories GmbH, Pasching, Austria) and centrifuged. Finally, the cells were washed once with KRG and resuspended in the same buffer at a density of 2×10^7^/ml. Calcium measurements were carried out in a Perkin Elmer fluorescence spectrophotometer (LC50), with excitation wavelengths of 340 nm and 380 nm, an emission wavelength of 509 nm, and slit widths of 5 nm and 10 nm, respectively. The transient rise in intracellular calcium is presented as the ratio of fluorescence intensities (340 nm: 380 nm) detected. The measuring cuvette contained catalase (2000 U) to counteract inactivation of the chemoattractants by the MPO-H_2_O_2_-system [39].

### The Cellular Content of F-actin

The F-actin content in neutrophils was analyzed by staining with FITC-phalloidin. The cells were fixed with equal volumes of paraformaldehyde (4% w/v in PBS), permeabilized with Triton X-100 (0.1% W/V in PBS), and incubated with FITC-phalloidin according to the manufacturer’s instructions. The cellular content of F-actin was determined by flow cytometry using an AccuriC6 cytometer (Becton Dickinson, Mountain View, CA, USA).

## Results

### Receptor Hierarchy between FPRs and the Receptors for PAF (PAFR) and IL8 (CXCR1/2)

**Figure 1:**
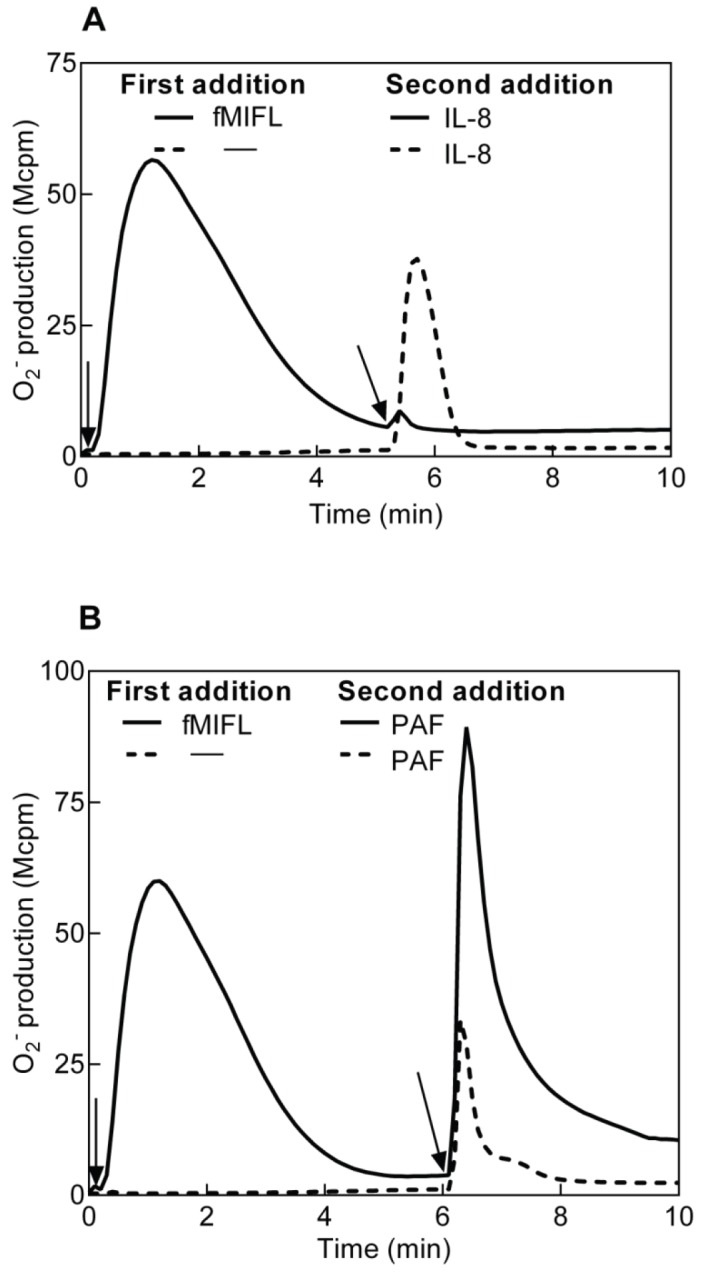
Receptor cross talk between neutrophil FPR1 and PAFR/CXCR1/2 determined as superoxide production. Human neutrophils desensitized with fMIFL were cross-desensitized to IL8 (**A**) but primed in their response to PAF (**B**). Neutrophils (10^5^ cells, 37°C) were first activated by the FPR1 specific agonist fMIFL (0.1 nM, added at time indicated by the arrows to the left) leading to receptor desensitization (solid lines in **A** and **B**). A second stimulus (**A**; IL8, 100 ng/ml, **B**; PAF, 100 nM) was added to the cells (solid lines) at the time point indicated by the arrows to the right. Activation of naïve (non-desensitized) neutrophils by IL8 (**A**) and PAF (**B**) was determined in parallel and is shown for comparison (broken lines). A representative experiment is shown, n>5. Abscissa, time of study (min); Ordinate, superoxide production (counts per minute×10^6^; Mcpm).

Formylated peptides are potent activators of neutrophil granulocytes, binding to 7TMRs of the FPR family [13], [14]. Neutrophils exposed to low nM concentrations of the FPR1-specific formylated peptide fMIFL respond by rapid activation of the NADPH-oxidase, resulting in release of superoxide anions (Fig. 1). The fMIFL-induced response is transient and terminates in less than 5 minutes after which the cells become non-responsive to a new challenge with the same agonist (data not shown and [39]). The fMIFL-stimulated cells have thus been transferred to an FPR1 desensitized state (FPR1_des_). The FPR1 has been shown to communicate with the IL8 receptors CXCR1/2 [40]. Accordingly, FPR1 activation led to desensitization not only of FPR1 but also of CXCR1/2; no superoxide release was induced when IL8 was added to FPR_des_ neutrophils (Fig. 1A). This cross talk was hierarchial (uni-directional) shown by that FPR1 was not desensitized by pre-stimulation of cells with IL8 (data not shown). The FPR_des_ cells were desensitized also to the lipid chemoattractant LTB_4_ (data not shown).

The molecular mechanism behind heterologous receptor desensitization between FPR1 and CXCR1/2 has been attributed to hierarchical signaling downstream of the two receptors [28]. Such hierarchical receptor desensitization is however not valid for the PAFR. When IL-8 was replaced by PAF as the trigger of superoxide anion release from FPR1_des_ cells, the cells were fully responsive (Fig. 1B). In fact, the PAF response in the FPR1_des_ cells was actually primed; the superoxide response was stronger and more persistent than the PAF response in naïve cells (Fig. 1B, 2B). Similar results were obtained with neutrophils desensitized to another FPR1 agonist (fMLF) or an FPR2 agonist (WKYMVM); also these cells were heterologously desensitized to IL8 but primed when challenged with PAF (data not shown).

**Figure 2:**
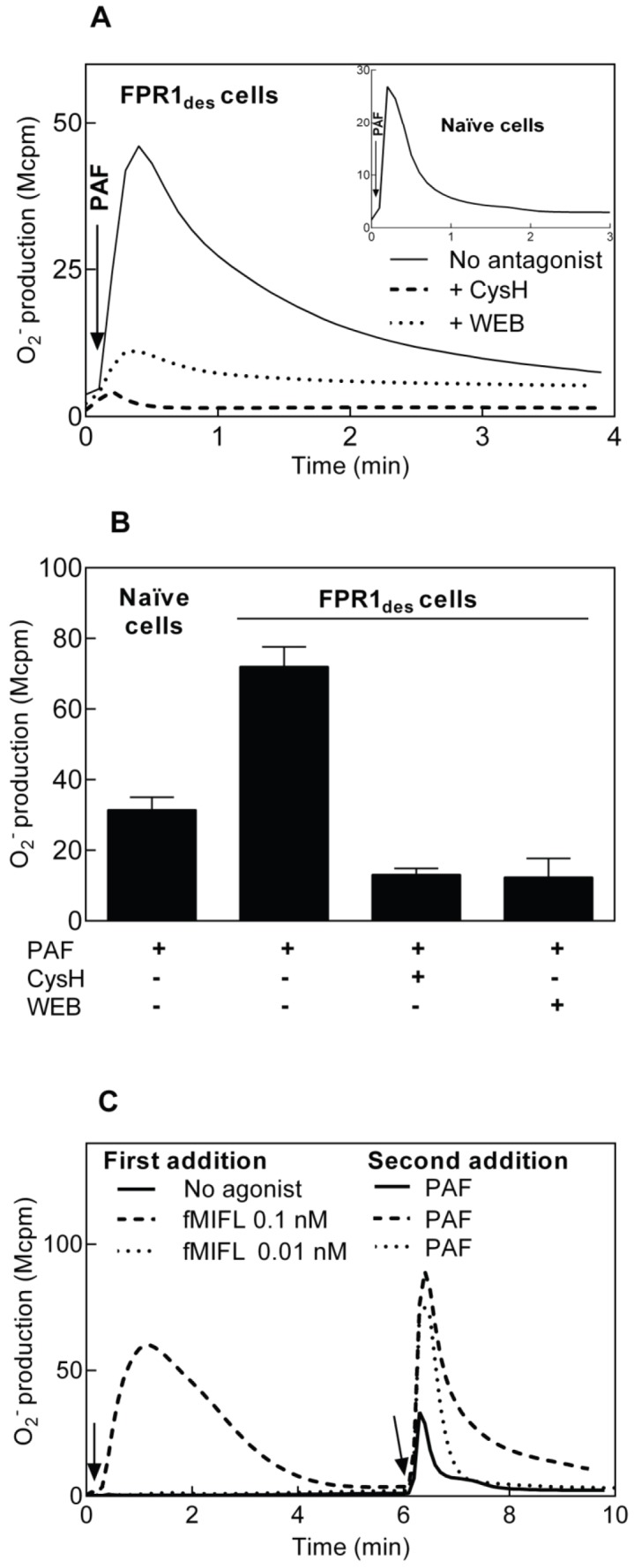
Receptor cross talk from the PAFR induces reactivation of FPR1_des_. Human neutrophils (10^5^) were desensitized with the FPR1 agonist fMIFL (0.1 nM) as described in Figure 1. (**A**) The FPR1_des_ neutrophils were activated with PAF (100 nM, added at time indicated by arrow; solid line). The involvment of FPR1 and PAFR in the PAF-induced response was examined by addition of cyclosporin H (1 µM, FPR1 antagonist, broken line) or WEB2086 (1 µM, PAFR antagonist, dotted line) at 3 min prior to PAF addition. For comparison, the oxidative response to PAF in naïve neutrophils is shown (inset). A representative experiment is shown, n>5. Abscissa, time of study (min); Ordinate, superoxide production (counts per minute×10^6^; Mcpm). (**B**) Inhibition of the PAF-induced response in FPR1_des_ cells by cyclosporin H (1 µM, FPR1 specific antagonist) or WEB2086 (1 µM, PAFR antagonist) shown as mean peak values ±SEM of the responses (Mcpm, n = 5 for WEB2086, n = 19 for control, cyclosporine H). The PAF induced response in naïve neutrophils is shown for comparison (n = 19). (**C**) Human neutrophils (10^5^) were activated/desensitized with different concentrations of the FPR1 agonist fMIFL (added at time indicated by arrow to the left). The neutrophils were then activated with PAF (100 nM final concentration, added at time indicated by arrow to the right). For comparison, a PAF-induced response in naïve neutrophils is shown (solid line). A representative experiment is shown, n>5. Abscissa, time of study (min); Ordinate, superoxide production (counts per minute×10^6^; Mcpm).

We conclude that agonist binding to FPRs induced homologous desensitization of the occupied receptor as well as heterologous desensitization of the receptors for IL8 and LTB4. In contrast, agonist binding of FPRs potently primed the response to PAF.

### Receptor Specific Antagonists Inhibit the Responses Induced by PAFR and FPR Agonists in Naïve Neutrophils

To ellucidate the molecular basis for the cross talk between FPRs and PAFR described above, we used receptor specific inhibitors (Table 1). As expected, the PAFR antagonist WEB2086 completely and selectively abolished the release of superoxide upon PAF stimulation, demonstrating that PAFR is responsible for the PAF-induced activation of human neutrophils (Fig. S1). It should be noted that PAF is a fairly potent stimulus with an EC_50_ of ≈ 500 nM (for comparison, the fMLF EC_50_ = 20 nM and the fMIFL EC_50_ = 0.2 nM). The FPR1 specific antagonist cyclosporin H abolished the release of superoxide upon fMIFL (or fMLF) stimulation and the FPR2 specific inhibitor PBP10 totally inhibited the superoxide release induced by the FPR2 specific agonist WKYMVM (Fig. S1). At the concentrations used, there were no cross-inhibitory effects of the PAFR antagonist on the fMIFL- or WKYMVM-induced neutrophil responses, and the FPR blockers were without effects on the PAF-induced response.

**Table 1:** Characteristics of the receptor antagonists used.

	CyclosporinH	PBP10	WEB 2086
**Basic description**	*A cyclic undeca-peptide, cyclosporin with* *more specific effects than other FPR1* *antagonists*	*A ten amino acid long peptide derived from* *a PIP_2_-binding domain of gelsolin and* *linked to rhodamine*	*A synthetic small molecule, potent inhibitor of PAF induced activity in platelets*
**Effects on FPR1**	inhibits neutrophil superoxide productionby more than 90% when induced byfMLF and fMIFL = specific for FPR1	primes neutrophil superoxide productionslightly when induced by fMLF and fMIFL	no effect on neutrophil superoxide production induced by fMLF and fMIFL
**Effects on FPR2**	no effect on neutrophil superoxideproduction induced by WKYMVM	inhibts neutrophil superoxide productionby more than 90% when triggered byWKYMVM = specific for FPR2	no effect on neutrophil superoxide production induced by WKYMVM
**Effects on PAFR**	no effect on neutrophil superoxideproduction induced by PAF	no effect on neutrophil superoxideproduction induced by PAF	inhibits neutrophil superoxide production by more than 95% when induced by PAF = specific for PAFR

### PAF Triggers a Reactivation of FPR1_des_ in Neutrophils

The antagonist effects were next determined in FPR1_des_ cells activated by PAF. Addition of the PAFR antagonist WEB2086 to FPR1_des_ neutrophils 1 min prior to PAF stimulation resulted, as expected, in a significant inhibition of the PAF response (Fig. 2A & B), showing that the response requires signaling through the PAFR. Unexpectedly, however, the PAF-induced response was largely inhibited also by the FPR1 specific antagonist cyclosporin H, when added 1 min prior to PAF stimulation (Fig. 2A & B). This implies that the PAF-triggered response in FPR1_des_ cells involves also activation of FPR1, i.e., there is a cross talk between the two receptors.

We next tested whether the reactivation effect was dependent on agonist occupancy of FPR1. When neutrophils were desensitized by 0.1 nM fMIFL at 15°C [41] and then diluted to a final concentration of 1 pM of the peptide, the cells could not be reactivated by PAF (data not shown). In contrast, if such FPR_des_ cells were diluted without reducing the fMIFL concentration, PAF-induced reactivation was intact (data not shown). This indicates that PAF-induced reactivation of FPR1_des_ neutrophils relies on a continual occupancy of FPR1 by fMIFL present in the surrounding medium. Furthermore, a cross talk signal induced by PAF was evident even when the concentration of fMIFL (used to desensitize FPR1) was as low as 10 pM, a concentration that in it self is too low to induce any respiratory burst activity in naïve neutrophils (Fig. 2C). Comparing the “pure” PAF response in FPR1_des_ cells, i.e., the response measured in the presence of the FPR1 antagonist cyclosporin H, with the PAF-induced response in naïve neutrophils, revealed a substantially lower response in the FPR1_des_ cells (Fig. 2A inset and 2B). The EC_50_ value for PAF was, however, the same (around 500 nM) between the naïve and FPR1_des_ cells.

The PAF-induced reactivation phenomenon was not exclusive for FPR1 but was seen also for FPR2. The PAF induced response in FPR2_des_ cells (desensitized with WKYMVM) was blocked by the FPR2 specific inhibitor PBP10 (Fig. S2), in analogy with the results for FPR1_des_ cells. The reactivation of FPR2_des_ cells by PAF was FPR2 specific and did not engage FPR1 (cyclosporin H was without any effect; data not shown). Also desensitized C5aR could be reactivated by PAF, even though the response was very low, part of the PAF induced response in C5aR_des_ cells was sensitive to a C5aR antagonist (data not shown).

We next reversed the order in which the stimuli were added. Cells were first stimulated with PAF to generate PAFR_des_ neutrophils, after which the cells were activated with FPR1 or FPR2 agonists. The PAFR_des_ cells were fully responsive to both FPR agonists, and both responses were completely inhibited by the specific inhibitors cyclosporin H and PBP10, respectively (Fig. S3 and data not shown). The PAFR antagonist WEB2086 was however completely without effect on the responses triggered by fMIFL or WKYMVM in PAF_des_ cells (Fig. S3 and data not shown). The receptor cross talk is, thus, highly regulated and restricted to one direction, i.e., reactivation signals are only transmitted from the PAFR to the FPRs and not vice versa.

In addition to PAF, the PAFR recognizes the more stable PAF analogue mcPAF as well as the PAF precursor lysoPAF [42], which were examined for capacity to trigger the cross talk and reactivation of the FPR_des_. The mcPAF and lysoPAF induced a similar receptor cross talk and FPR1_des_ reactivation as PAF; i.e., the neutrophil NADPH-oxidase activity in FPR1_des_ cells triggered with mcPAF or lysoPAF was substantially inhibited by the FPR1 antagonist cyclosporin H (Fig. S4).

Taken together, our data clearly reveal a novel form of receptor cross talk from PAFR to FPR, leading to reactivation of desensitized FPRs.

### The PAF-induced Rise in Intracellular Ca^2+^ in FPR_des_ Neutrophils is not Inhibited by Cyclosporin H

**Figure 3:**
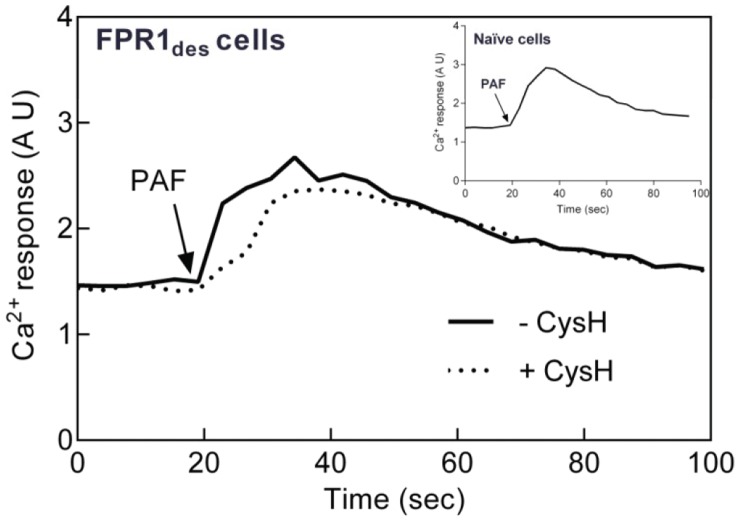
Intracellular Ca^2+^ response triggered upon reactivation of FPR1_des_ by PAF is not cyclosporin H sensitive. FPR1_des_ neutrophils (desensitized with 0.1 nM fMIFL) loaded with Fura-2 (2×10^6^/ml) were activated by PAF (1 nM final concentration) in the absence (solid line) or presence (broken line) of the FPR1 specific antagonist cyclosporin H (1 µM added 30 sec before PAF). The changes in fluorescence were followed using dual excitation of Fura-2 at 340 and 380 nm, respectively, with an emission wavelength of 510 nm. For comparison, a PAF-induced intracellular Ca^2+^ response is shown for naïve neutrophils (inset). A representative experiment is shown, n = 3. Abscissa, time of study (sec); Ordinate, relative change in ^hello^Ca^2+^]_i_ (arbitrary units, AU).

When 7TMR agonists bind their receptors, one of the very early signals generated is a rise in the cytosolic concentration of free Ca^2+^, achieved through emptying of intracellular Ca^2+^ stores. Consequently, naïve cells responded by transient increases in Ca^2+^ to both fMIFL (Fig. S6) and PAF (Fig. 3), effects that were completely blocked by cyclosporine H and WEB2086, respectively (data not shown). A rise in intracellular Ca^2+^ was also induced by PAF when added to FPR_des_ cells (Fig. 3). In contrast to the oxidative response, this Ca^2+^ response was not affected by cyclosporin H (Fig. 3), demonstrating that it is independent of FPR1.

When measuring activation of the NADPH-oxidase, the FPR_des_ cells were primed to PAF, giving a substantially increased oxidative response as compared to PAF-stimulated naïve cells. With regard to the Ca^2+^ response induced by PAF in FPR_des_ cells the magnitude was not elevated but rather decreased as compared to the PAF response in naïve neutrophils (Fig. 3 inset).

Taking these data together, we conclude that two signaling pathways are triggered by PAF in FPR_des_ neutrophils, one FPR-dependent signal that triggers oxidase activation and another, FPR-independent signal that leads to an intracellular Ca^2+^ increase.

### Opposite Effects of CalyculinA on Naïve and Desensitized Neutrophils

**Figure 4:**
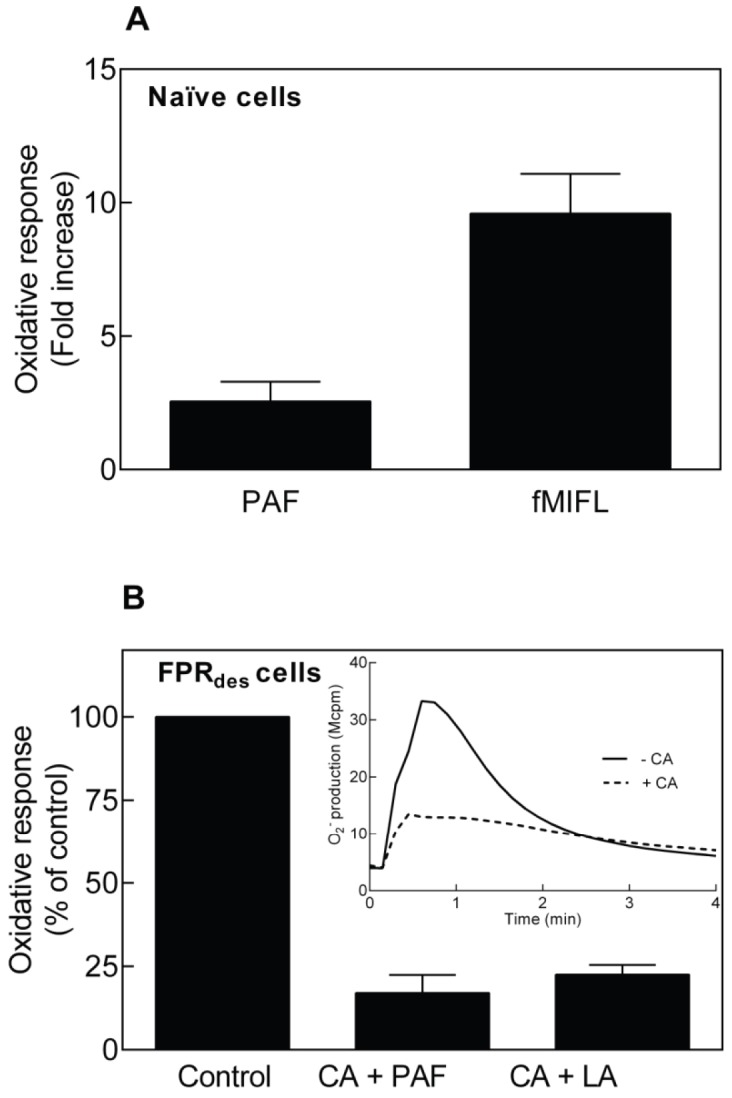
Phosphatase inhibition by CalyculinA has both inhibitory and priming effects on the neutrophil NADPH-oxidase response. (**A**) Human neutrophils were incubated without or with CalyculinA (CA; 60 nM) at 37°C for 10 min prior to stimulation with PAF (100 nM) or fMIFL (0.1 nM), and the release of superoxide anions was recorded. The graph shows ratios of superoxide production induced by PAF or fMLF between samples with and without calyculin A (fold increase, mean ±SEM; n = 5). (**B**) FPR1_des_ neutrophils (desensitized with 0.1 nM fMIFL) were incubated at 37°C for 10 min without (control and inset, solid line) or with CalyculinA (CA, 50 nM; inset, broken line). The cells were then stimulated with PAF (100 nM) or latrunculin A (100 ng/ml final concentration) and the release of superoxide anions was recorded. A representative experiment for PAF stimulation is shown in the inset. The stimulus-induced responses in the CalyculinA treated FPR1_des_ neutrophils are expressed as percent of non-treated controls and is given as means ±SEM (n = 8).

Phosphatase inhibition has been suggested to reduce binding of ligand-occupied FPRs to the actin cytoskeleton [32], a process known to limit/terminate the response triggered by FPRs [19]. Accordingly, phosphatase inhibitors have earlier been shown to prime cells to FPR1 agonists [32], [43]. CalyculinA is a phosphatase inhibitor that selectively inhibits the serine/threonine phosphatases PP1 and PP2A. We investigated the effect of CalyculinA on the PAF-induced oxidative responses of naïve and FPR_des_ neutrophils. We first confirmed that CalyculinA primes naive cells to FPR1 stimulation and in addition we found that also the PAF induced response in naïve neutrophils was primed (Fig. 4A). CalyculinA had no direct effect on the oxidase activity in naïve cells besides priming. We next investigated the effect of CalyculinA on the cross talk between the PAFR and FPR1. We found that CalyculinA blocked the PAF-induced reactivation of FPR_des_ cells (Fig. 4B), suggesting that serine/threonine phosphatases are involved in the PAF-induced cross talk signaling leading to reactivation of FPR_des_.

### Cytoskeleton-disrupting Agents Trigger a Reactivation of FPR_des_ that in Some Respects Resembles that of PAF

Agonist-binding rapidly transfers FPR to a non-signaling (FPR_des_) state and as mentioned above, coupling of ligand-receptor complexes to the actin cytoskeleton has been suggested to play a major role in the termination of signaling and desensitization process [17], [32]. The two drugs latrunculinA and cytochalasinB both disrupt the actin cytoskeleton in cells by interfering with the polymerization of filamentous (F-)actin during actin remodeling [44]. Accordingly, the presence of latrunculinA or cytochalasinB results in an increased and prolonged response when naïve neutrophils are activated by formylpeptides [45] or PAF (Fig. S5; Table 2).

**Table 2:** Characteristics of cytoskeleton interfering drugs used.

	LatrunculinA (50 ng/ml)	CalyculinA (60 nM)
**Basic description**	*A toxin that binds actin monomers and interferes with their* *addition to filamentous actin and by that the cytoskeleton* *is disrupted*	*A naturally occurring serine/threonine phosphatase inhibitor that increase the level of phosphorylation and inhibits binding of occupied receptors to the cytoskeleton*
**Effects on FPR1**	augments neutrophil superoxide production inducedby fMLF and fMIFL	augments neutrophil superoxide production induced by fMLF and fMIFL
**Effects on FPR2**	augments neutrophil superoxide production inducedby WKYMVM	augments neutrophil superoxide production induced by WKYMVM
**Effects on PAFR**	augments neutrophil superoxide production induced by PAF	augments neutrophil superoxide production induced by PAF

**Figure 5:**
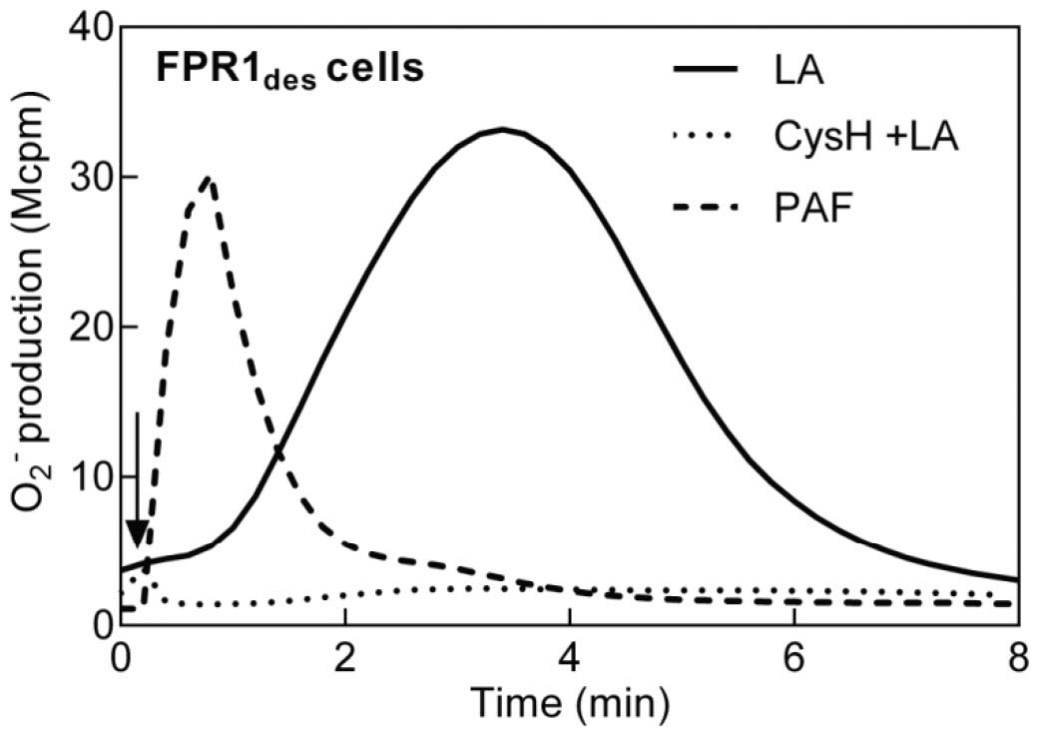
The cytoskeleton disrupting agent latruculin A induces reactivation of FPR1_des_. Latrunculin A (100 ng/ml) was added to FPR1_des_ neutrophils (10^5^ cells; desensitized with 0.1 nM fMIFL) in the absence (solid line) or presence (dotted line) of cyclosporin H (1 µM, FPR1 specific antagonist, added 1 min before latrunculin A) and the release of superoxide anions was determined. For comparison, a PAF-induced reactivation of FPR1_des_ neutrophils is included (dashed line). A representative experiment is shown, n>5. Abscissa, time of study (min); Ordinate, superoxide production (counts per minute×10^6^; Mcpm).

Similar to the reactivation of FPR_des_ cells by PAF, addition of latrunculinA to these cells induced a pronounced, cyclosporin H-sensitive, reactivation of the NADPH-oxidase, although with a different time course (Fig. 5). LatrunculinA-induced reactivation was induced also in FPR2_des_ cells, and PBP10 abolished this response completely (data not shown). Taken together, our data show that FPR_des_ reactivation can be achieved not only by PAF, but also by disruption of the actin cytoskeleton.

No direct activation was obtained by latrunculinA or cytochalasinB when added alone to naïve neutrophils (data not shown), and no superoxide release was obtained from PAFR_des_ cells upon the addition of the inhibitors (data now shown).

### PAF- and latrunculinA-induced Reactivation of FPRs Display Similarities in Signaling

As stated above, the PAF-induced NADPH-oxidase activation in FPR1_des_ cells is not associated with a cytosolic Ca^2+^ transient. Similarly, superoxide production induced by reactivation of FPR_des_ cells by latrunculinA occurred without any rise in intracellular Ca^2+^ (Fig. S6 inset). The FPR_des_ reactivation leading to superoxide production is thus not associated with any activation of the PLC/IP_3_ signaling route that leads to an emptying of the intracellular Ca^2+^ stores.

Also in agreement with the PAF-induced reactivation of FPR_des_, the latrunculinA-induced reactivation was inhibited by CalyculinA (Fig. 5). Taken together, these data indicate that similar signaling pathways are operating when FPR_des_ are reactivated by PAF and by disruption of the cytoskeleton.

### PAF-induced Reactivation of FPR_des_ Occurs Regardless of Receptor Uncoupling from the Cytoskeleton

**Figure 6:**
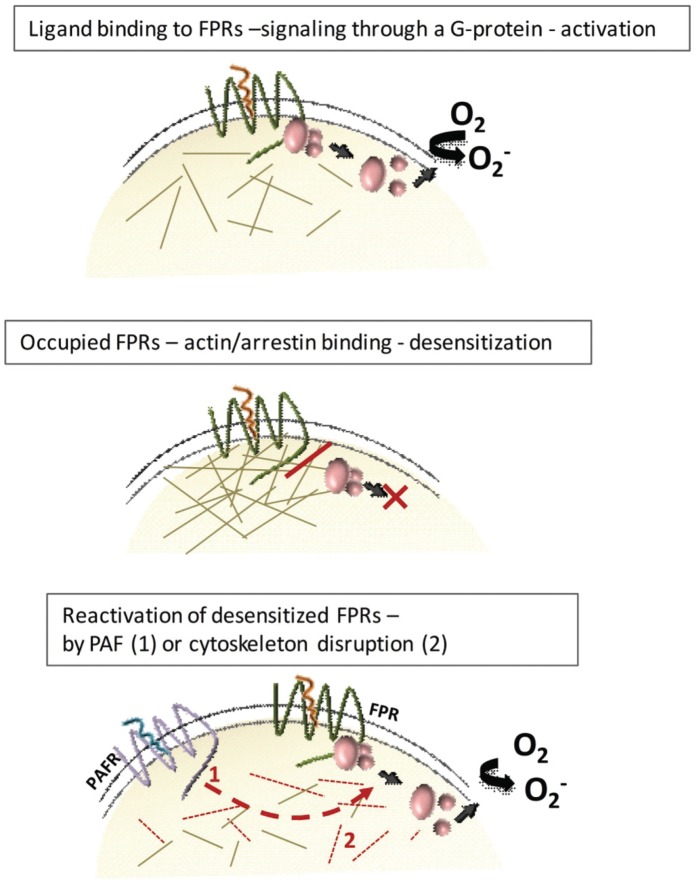
Model for FPR activation, desensitization and reactivation. **A)** The agonist-occupied FPR activates a G-protein and the second messengers generated activate the electron-transporting NADPH-oxidase that reduces oxygen to superopxide anion. The signaling state of the receptor is fairly short lived. **B**) The agonist-occupied receptor is desensitized and the functional response is terminated. This non-signaling state is hypothetically achieved through a physical separation of the receptor-ligand complex from the G-protein, made possible by binding of actin polymers and/or arrestin molecules to the receptor. **C**) The desensitized FPR is reactivated by signals generated when PAF binds to its neutrophil receptor (arrow, 1). Reactivation of the desensitized FPR is achieved also with cytoskeletal inhibitors, (shorter filaments, 2), suggesting a mechanism for reactivation that involves uncoupling of the receptor-ligand complex from the cytoskeleton. The described cross talk is hierarchial and unidirectional.

**Figure 7:**
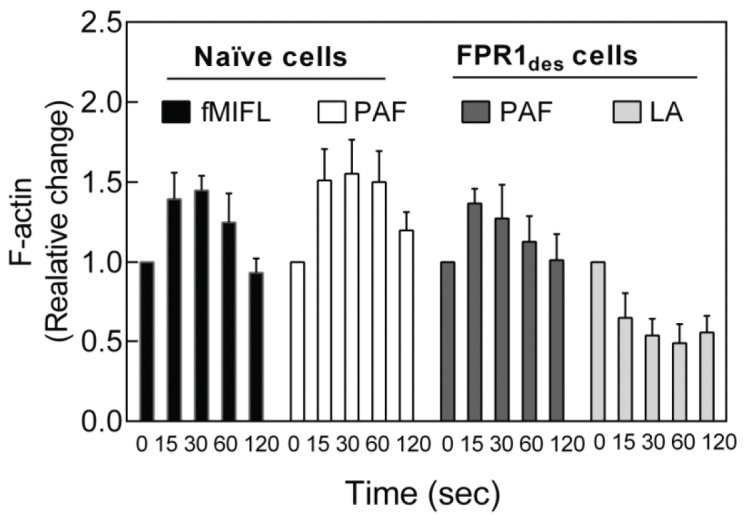
PAF induces actin polymerization in both naïve and FPR1_des_ neutrophils. Human neutrophils (naïve or FPR1_des_) were activated with a receptor agonist or latrunculin A and the change in polymerized actin was determined att different time points (15 to 120 sec) after activation. Naïve neutrophils were activated by PAF (100 nM) or fMLF (0.1 nM) and FPR1_des_ neutrophils were reactivated by PAF (100 nM) or latrunculin A (200 ng/ml). The stimulation at indicated time points was terminated by adding ice cold paraformyldehyde (final concentration 2%) to the cells. The amount of polymerized actin was determined by flow cytometry after phalloidin staining and compared to the amount of actin at time zero before activation. The values are shown as mean ratio ± SEM; n = 3.

Separation of ligand-receptor complexes from signaling G-proteins through a direct interaction of the occupied receptors with the actin cytoskeleton could form the molecular basis for both receptor desensitization and reactivation (see the model presented in Fig. 6). The similarity between PAF and inhibitors of actin polymerization in reactivation of FPR_des_ promoted us to investigate the effects of PAF on actin polymerization in FPR_des_ cells. As measured by phalloidin staining, PAF induced a rapid and transient polymerization of actin in both naïve and FPR_des_ neutrophils, and the levels were of similar magnitude (Fig. 7). The reactivation of FPR_des_ neutrophils by latrunculinA was associated with reduced levels of actin polymerization, as expected (Fig. 7).

**Figure 8:**
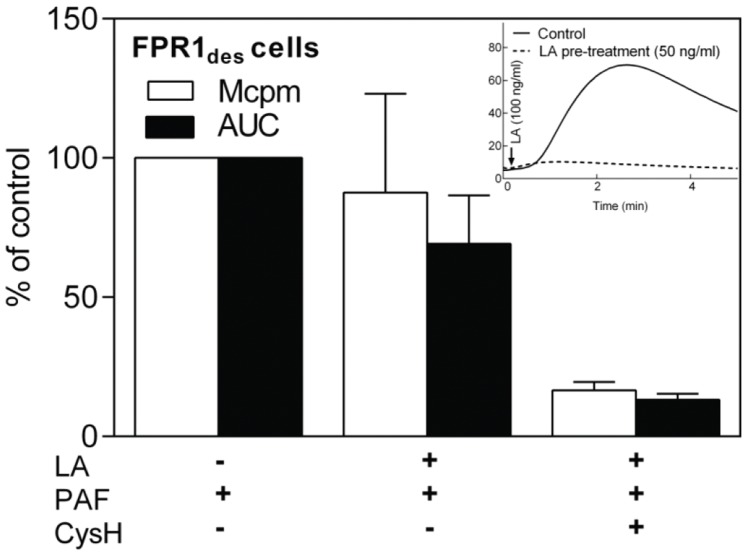
PAF activates FPR1_des_ neutrophils also in the presence of latrunculinA. Human FPR1_des_ neutrophils were incubated in the absence or presence of latunculinA (LA, 50 ng/ml) and after return of the NADPH-oxidase activity to background levels (after around 20 min; not shown in the figure) the cells were activated with PAF (100 nM) and the measurement of oxidase activity was started. In some experiments, cyclosporinH (CA, 1 µM) was added to the cells just prior to PAF. The response induced was sensitive to this FPR1 specific antagonist. The results are expressed as peak response (Mcpm, open bars) and total production (area under curve; AUC, filled bars) in percent of control (PAF-induced peak response in FPRdes in the absence of LA and CA; mean±SEM, n = 3). The FPR1_des_ neutrophils treated with latrunculin A (50 ng/ml) could not be reactivated by additional latrunculin A (100 ng/ml, inset, dotted line). For comparison, reactivation of control cells (FPR1_des_ neutrophils without latrunculin A pre-treatment, solid line) is shown.

The oxidative reactivation response induced by latrunculinA in FPR_des_ cells declines slowly (Fig. 5) and when the activity has returned to basal level, the cells are refractory to further stimulation/reactivation by another dose of either fMIFL or latrunculinA (Fig. 8, inset, and data not shown). This suggests that the actin cytoskeleton is fully disrupted in the latrunculinA treated FPR_des_ cells. However, addition of PAF to latrunculinA-treated FPR_des_ cells resulted in a new burst of superoxide, a response that was inhibited by cyclosporin H (Fig. 8). This strongly suggests that the cross talk signals generated by PAF to trigger reactivation of FPR_des_ is transmitted in an actin-independent manner.

In conclusion, although the reactivation of FPRdes cells by PAF and cytoskeleton-disrupting agents share signaling pathways, the disruption of actin per se is not part of the PAF-induced signaling leading to reactivation.

## Discussion

Neutrophils as well as most other cell types express many different 7TMRs and one specific ligand–receptor pair does not generally or necessarily operate alone. On the contrary, co-expressed receptors have the ability to communicate with one another. Such receptor cross talk can involve i) a direct physical interaction between identical or different receptors, ii) receptor phosphorylation that “spills over” from one occupied receptor to another, and iii) cross talk of downstream signaling events [46]. We now describe a novel receptor cross talk mechanism in neutrophils, unique in that the signals generated by one 7TMR transfer another receptor from a desensitized (non-signaling) state back to an actively signaling state. To our knowledge, this is the first description of such a unique cross talk between two GPCRs.

Our full understanding of the mechanisms behind the described receptor reactivation, is prohibited by the general lack in basic knowledge regarding termination of signaling from an occupied FPR. Although we have made several attempts to gain knowledge on the molecular mechanisms that underlie the discussed desensitization and reactivation phenomena in neutrophils, we can at present only speculate on their composition and function. Much work remains to be done before we can fully understand not only the cross talk at a molecular level but also its biological significance. Possible mechanisms, operating at multiple levels are discussed below and some of the ideas put forward should be regarded as mere speculations.

The FPRs and the PAFR share many features but there is at least one fundamental difference between the desensitized state of these two receptor types; the desensitized FPRs can be reactivated while the PAFR cannot. This suggests that different regulatory mechanisms for desensitization are operating. Reactivation of FPR_des_ is hardly directly linked to receptor internalization and recycling since reactivation can be achieved following an initial interaction of neutrophils and the FPR ligand at a temperature (15°C) that allows receptor desensitization but is too low to permit receptor internalization.

Currently the foremost accepted model for desensitization of GPCRs highlights the role of β-arrestin-receptor binding as the basis for termination of signaling. Even though FPRs bind arrestin [15] this mechanism seeems to be of minor importance for the termination of FPR sigaling [16]. Instead we and others have proposed a direct binding of the signaling receptor-ligand complex to the actin cytoskeleton (Fig. 6) as the terminating event. According to this model, the cytoskeleton physically separates the ligand-receptor complex from the signaling G-protein, terminating downstream transduction of signals [18], [47]. Experimental support for this mechanism is based on pharmacological inhibition of actin polymerization which prolongs signaling from occupied FPRs, and our data on receptor reactivation induced by latrunculinA also fits this model like a glove. There must, however, be mechanism(s) apart from actin dynamics that terminate the signaling since, i) signaling from neutrophil GPCRs (including both FPRs and PAFR) is terminated also when the cytoskeleton is disrupted by inhibitors of actin polymerization (i.e., latrunculinA and cytochalasinB), and ii) the desensitized PAFR is not reactivated when the cytoskeleton is disrupted.

With regard to involvement of cytoskeleton uncoupling as basis for the PAF-induced reactivation of FPR_des_ cells discussed in this study, this is an attractive hypothesis as there are valid similarities between the reactivation responses induced by latrunculinA and PAF (e.g., both responses are inhibited by the phosphatase inhibitor CalyculinA), However, PAF reactivated FPR_des_ also when the actin cytoskeleton had been disrupted, and our data showing no net reduction of polymerized actin during PAF-induced FPR1_des_ reactivation are also in opposition to such a model.

We show that FPR/PAFR activation as well as FPR_des_ reactivation depend on cellular phosphorylation levels. CalyculinA primed the direct activation of the FPRs in naïve cells while reactivation induced by PAF in FPR_des_ cells was inhibited. Previous studies in naïve neutrophils have shown that FPR1, as well as many other proteins, are phosphorylated upon agonist binding. This phosphorylation is thought critical for receptor internalization and desensitization, as well as for β-arrestin binding [10], [48], [49], [50]. We have earlier suggested that the priming effect induced in naïve neutrophils by phosphatase inhibition is due to decreased binding of occupied receptors to the cytoskeleton [32]. It is however hard to fully fit the results on both naïve cells and FPR_des_ neutrophils into this model. Clearly, there might be several other basic mechanisms behind the phenomena described and at present we cannot distinguish whetherthe phosphorylation level affects one or the other of the two receptors involved, some of the unknown downstream signaling molecules, and/or the direct assembly and function of the NADPH-oxidase. Inhibition of phosphatases will lead to an increased level of phosphorylation irrespectively if the receptors trigger activation of CalyculinA sensitive phosphatases or not, and we know virtually nothing about the identity of the protein(s) that prime naïve cells and inhibits desensitized cells.

The protein β-arrestin, initially identified as a mediator for GPCR desensitization and internalization, has not been studied in primary neutrophils. Recent research using other cell types has, however, drawn much attention to the very complex relationship between receptor binding of β-arrestin and downstream phosphorylation reactions and receptor as well as to its roles in signaling achieved by scaffolding of signaling proteins following receptor recruitment [51]. It is of particular interest that β-arrestins bind a number of actin assembly proteins and thus may play a requisite role in reorganization of the actin cytoskeleton [52]. The precise mechanisms by which this regulation of actin reorganization is achieved, and the role this has as a regulatory pathway in neutrophils is not known. In our attempt to understand the signalings involved in FPR_des_ reactivation, we show that this process does not trigger a Ca^2+^ response, a feature necessary to the signaling pathways of most GPCRs. In relation to this it is interesting to note that many of the scaffold functions of β-arrestin occurs without any involvement of classical signaling G-proteins. Whether β-arrestins plays a role in FPR desensitization remains to be determined, together with the possible impact of multiple signaling β-arrestin scaffolds in FPR_des_. The fact that the signaling route ultimately leading to reactivation of FPR_des_ bypasses the Ca^2+^ pathway will in the future direct our attention to cell models that express the two cross talking receptors in conjunction with a Ca^2+^ independent read-out system triggered by the reactivated receptor.

In summary, the data presented in this study provide evidence that PAF can modulate neutrophil functions, either directly or through a receptor cross talk with other receptors, and by this promote the neutrophil activation. These findings not only point to the possibility that PAF-mediated pathology may involve cross talk with other receptors that are reactivated by PAF stimulation, but also demonstrate that unique signaling pathways are utilized downstream of the PAFR, leading to priming and agonist-driven receptor reactivation. Clearly, more experiments are needed in the future in order to validate our hypothesis regarding the direct role of actin-depedent versus β-arrestin-mediated desensitization pathways. Also the involvement of β-arrestin scaffold-mediated signaling, and of so far unidentified signaling pathway(s) that may be linked in one way or another to the cell cytoskeleton, requires further study. Our data showing that FPR_des_ can be reactivated by PAF also when the actin cytoskeleton has been disrupted, strongly support the concept that FPR can be desensitized through an actin-independent pathway.

## Supporting Information

Figure S1 **Characterization of receptor specific antagonists for FPRs and PAFR in naïve neutrophils.** Naïve neutrophils (10^5^ cells) were incubated in the absence (solid lines) or presence (broken lines) of antagonist (WEB2086, 1 µM, a PAFR specific antagonist; cyclosporin H, 1 µM an FPR1 specific antagonist; PBP10, 1 µM an FPR2 specific antagonist) for 5 min at 37°C and were then activated with PAF (100 nM, upper panel), fMIFL (0.1 nM, middle panel), or WKYMVM (100 nM, lower panel). A representative experiment is shown, n>5. Abscissa, time of study (min); ordinate, superoxide production (counts per minute×10^6^, Mcpm).
(TIF)

Figure S2 **A PAFR-initiated cross talk induces reactivation of FPR2 in desensitized neutrophils.** Human neutrophils (10^5^) were desensitized with the FPR2 agonist WKYMVM (100 nM final concentration) and subsequently activated with PAF (100 nM final concentration, added at arrow). The involvment of FPR2 in the resulting PAF-induced superoxide production was examined by addition of the FPR2 antagonist PBP10 (1 µM, dotted line) 1 min before the addition of PAF. For comparison, a PAF-induced response in naïve neutrophils is shown (inset). Representative experiments are shown, n>5. Abscissa, time of study (min); Ordinate, superoxide production (counts per minute×10^6^, Mcpm).
(TIF)

Figure S3 **No reactivation is induced by fMIFL in PAFR_des_ neutrophils.** Human neutrophils (10^5^) were desensitized with PAF (100 nM final concentration). The desensitized neutrophils were activated with fMIFL (0.1 nM final concentration, added arrow; solid line). The involvement of FPR1 and PAFR in fMIFL-induced superoxide production was examined by addition of cyclosporin H (1 µM, FPR1 antagonist, dotted line) or WEB2086 (1 µM, PAFR antagonist, broken line) 1 min before addition of fMIFL. For comparison, a fMIFL-induced response in naïve neutrophils is shown (inset). A representative experiment is shown, n>5. Abscissa, time of study (min); Ordinate, superoxide production (counts per minute×10^6^, Mcpm).
(TIF)

Figure S4 **The PAF precursor lysoPAF and the stable analogue mcPAF both reactivate FPR1_des_ neutrophils.** Human neutrophils (10^5^) were desensitized with the FPR1 agonist fMIFL (0.1 nM final concentration). The desensitized neutrophils were activated with lysoPAF (**A**; 1 µM final concentration added at arrow; solid line) or mcPAF (**B**; 1 µM final concentration added at arrow; solid line). The involvement of FPR1 in the responses was examined by the addition of cyclosporin H (1 µM, FPR1 antagonist, broken lines) 1 min before addition of the agonist. For comparison, a lyso PAF- (A, inset) or mcPAF- (B, inset) induced response in naïve neutrophils is shown. The figures show representative experiments, n>5. Abscissa, time of study (min); Ordinate, superoxide production (counts per minute×10^6^, Mcpm).
(TIF)

Figure S5 **The PAF-induced neutrophil response is primed by inhibitors of actin polymerization.** Naïve human neutrophils were incubated at 37°C for 5 min with either Cytochalasin B (Cyt B, 5 µg/ml; grey bars) or latrunculin A (LA, 50 ng/ml; white bars). Control cells were incubated at the same conditions but in the absence of actin polymerization inhibitor. The cells were then activated with PAF (100 nM) and the release of superoxide was recorded continuously. Data are expressed as fold increase of peak values in treated cells as compared to non-treated controls (mean ± SEM; n = 3). The dashed line denotes the value expected in the absence of effect.
(TIF)

Figure S6 **Latrunculin A induces no increase in intracellular Ca^2+^ in FPR1_des_ neutrophils.** Intracellular Ca^2+^ canges was determined in Fura-2 loaded naïve and FPR1_des_ (0.1 nM fMIFL) neutrophils. Naïve neutrophils were activated by fMIFL (1 nM; solid line), and FPR1_des_ neutrophils were reactivated by latrunculin A (100 ng/ml; inset). The changes in fluorescence were followed using dual excitation at 340 nm and 380 nm, and an emission wavelength of 510 nm. Representative experiments are shown. Abscissa, time of study (min); Ordinate, relative change in [Ca^2+^]_i_.
(TIF)
