# Supplementary figures and images for: Reactivation of Desensitized Formyl Peptide Receptors by Platelet Activating Factor: A Novel Receptor Cross Talk Mechanism Regulating Neutrophil Superoxide Anion Production
Source: PLoS One. 2013 Mar 28;8(3):e60169. doi: 10.1371/journal.pone.0060169 (PMC3610682; doi:10.1371/journal.pone.0060169)

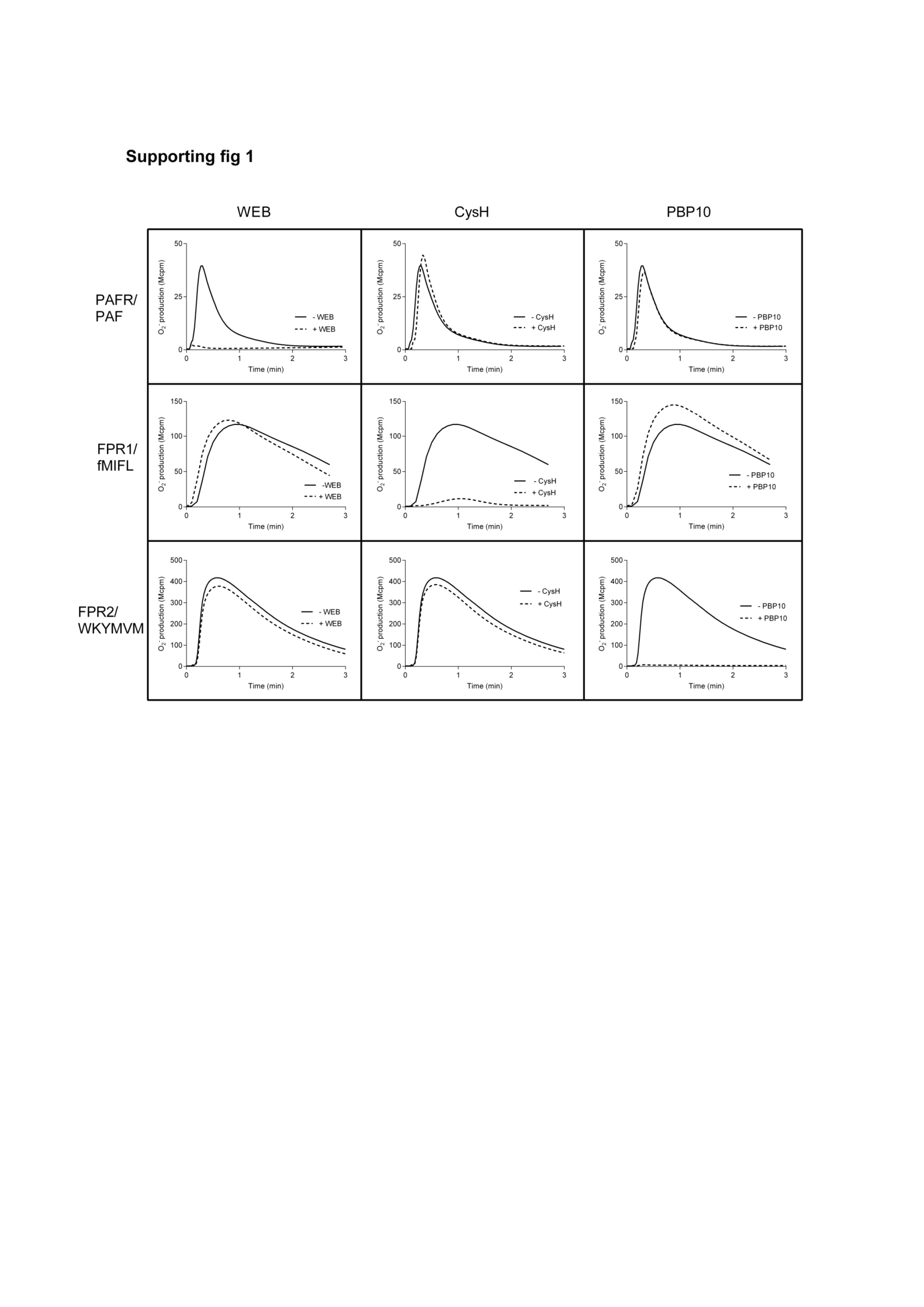

Supplement: Figure S1 — Characterization of receptor specific antagonists for FPRs and PAFR in naïve neutrophils. Naïve neutrophils (105 cells) were incubated in the absence (solid lines) or presence (broken lines) of antagonist (WEB2086, 1 µM, a PAFR specific antagonist; cyclosporin H, 1 µM an FPR1 specific antagonist; PBP10, 1 µM an FPR2 specific antagonist) for 5 min at 37°C and were then activated with PAF (100 nM, upper panel), fMIFL (0.1 nM, middle panel), or WKYMVM (100 nM, lower panel). A representative experiment is shown, n>5. Abscissa, time of study (min); ordinate, superoxide production (counts per minute×106, Mcpm). (TIF) [file pone.0060169.s001.tif]

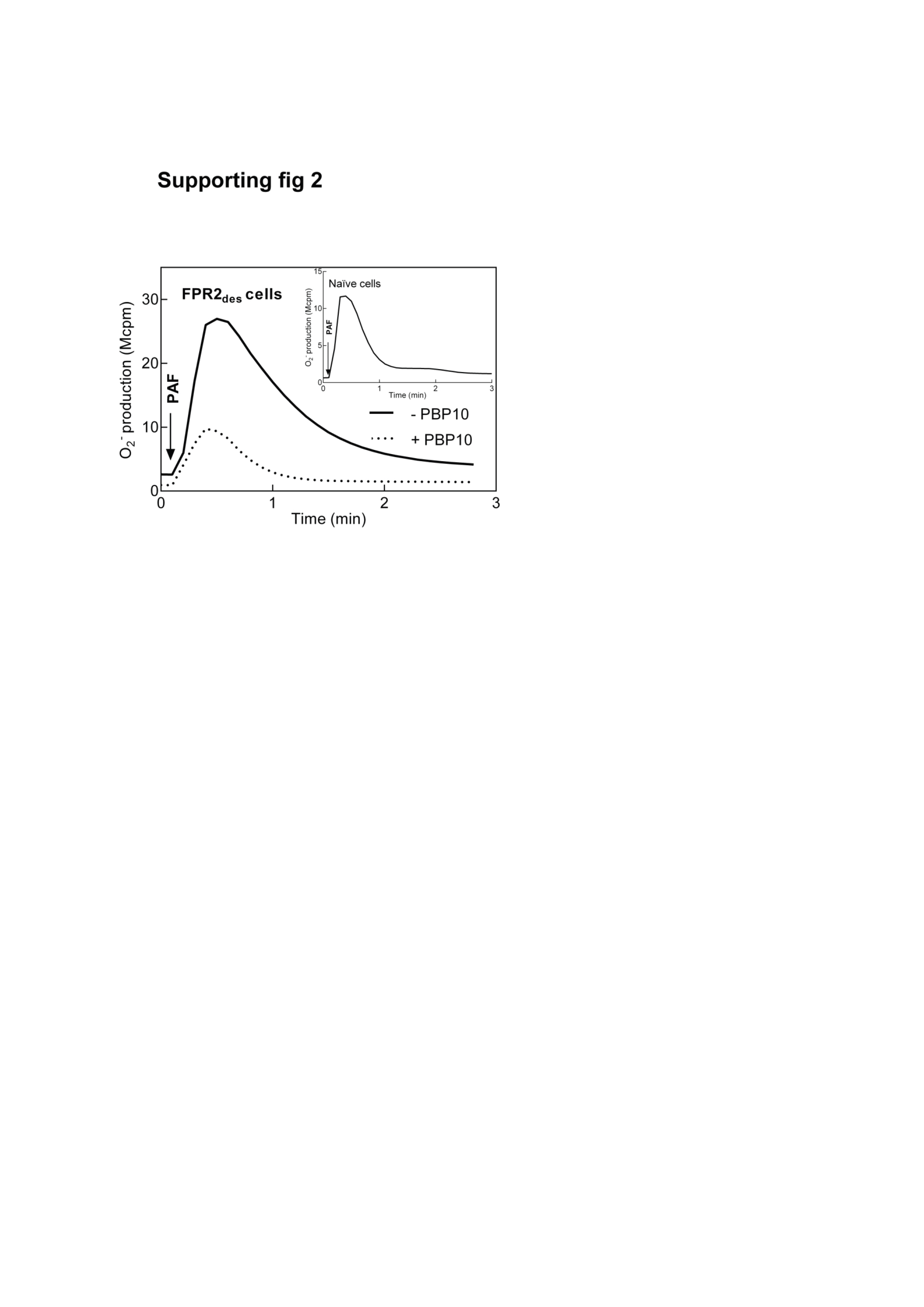

Supplement: Figure S2 — A PAFR-initiated cross talk induces reactivation of FPR2 in desensitized neutrophils. Human neutrophils (105) were desensitized with the FPR2 agonist WKYMVM (100 nM final concentration) and subsequently activated with PAF (100 nM final concentration, added at arrow). The involvment of FPR2 in the resulting PAF-induced superoxide production was examined by addition of the FPR2 antagonist PBP10 (1 µM, dotted line) 1 min before the addition of PAF. For comparison, a PAF-induced response in naïve neutrophils is shown (inset). Representative experiments are shown, n>5. Abscissa, time of study (min); Ordinate, superoxide production (counts per minute×106, Mcpm). (TIF) [file pone.0060169.s002.tif]

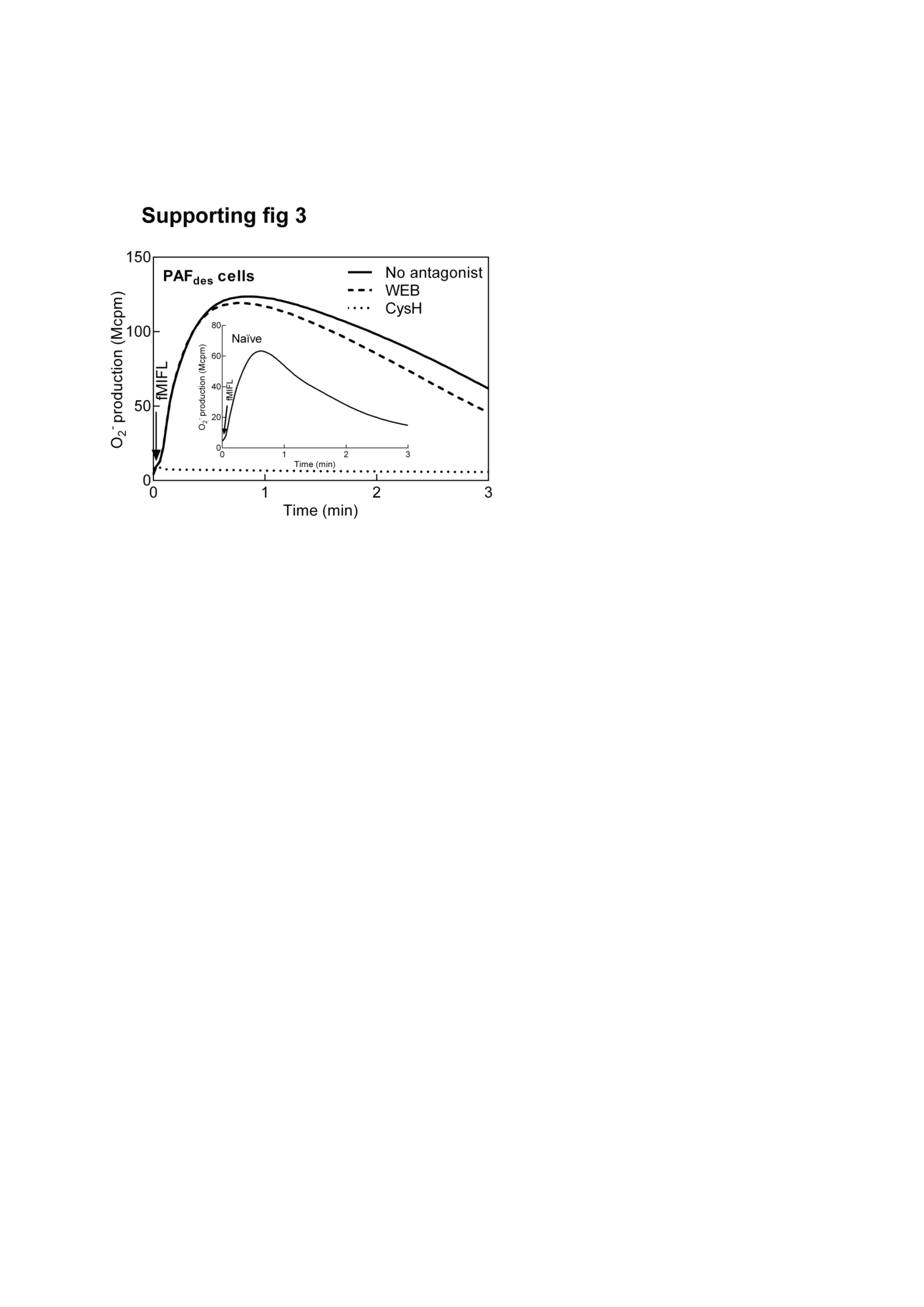

Supplement: Figure S3 — No reactivation is induced by fMIFL in PAFRdes neutrophils. Human neutrophils (105) were desensitized with PAF (100 nM final concentration). The desensitized neutrophils were activated with fMIFL (0.1 nM final concentration, added arrow; solid line). The involvement of FPR1 and PAFR in fMIFL-induced superoxide production was examined by addition of cyclosporin H (1 µM, FPR1 antagonist, dotted line) or WEB2086 (1 µM, PAFR antagonist, broken line) 1 min before addition of fMIFL. For comparison, a fMIFL-induced response in naïve neutrophils is shown (inset). A representative experiment is shown, n>5. Abscissa, time of study (min); Ordinate, superoxide production (counts per minute×106, Mcpm). (TIF) [file pone.0060169.s003.tif]

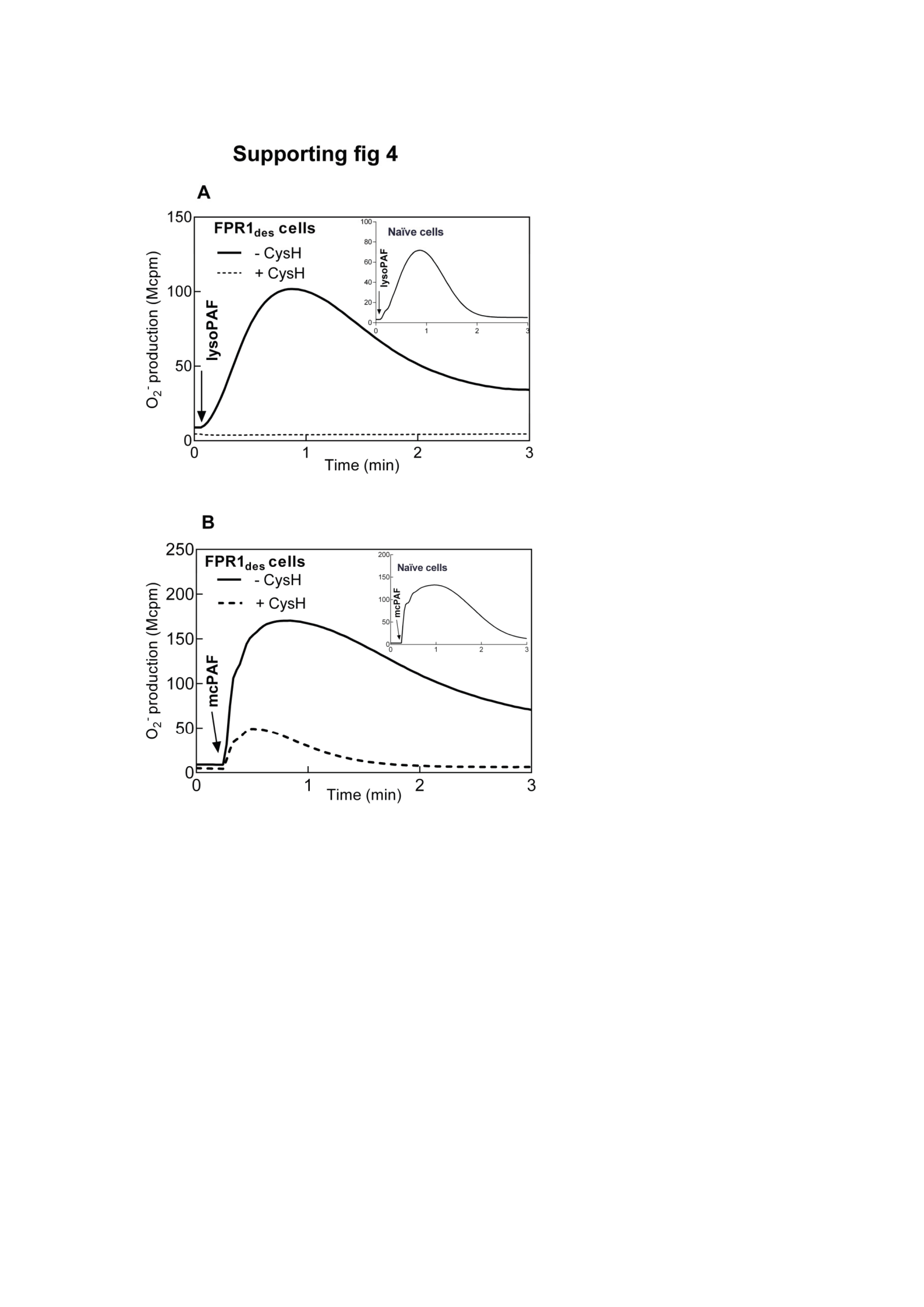

Supplement: Figure S4 — The PAF precursor lysoPAF and the stable analogue mcPAF both reactivate FPR1des neutrophils. Human neutrophils (105) were desensitized with the FPR1 agonist fMIFL (0.1 nM final concentration). The desensitized neutrophils were activated with lysoPAF (A; 1 µM final concentration added at arrow; solid line) or mcPAF (B; 1 µM final concentration added at arrow; solid line). The involvement of FPR1 in the responses was examined by the addition of cyclosporin H (1 µM, FPR1 antagonist, broken lines) 1 min before addition of the agonist. For comparison, a lyso PAF- (A, inset) or mcPAF- (B, inset) induced response in naïve neutrophils is shown. The figures show representative experiments, n>5. Abscissa, time of study (min); Ordinate, superoxide production (counts per minute×106, Mcpm). (TIF) [file pone.0060169.s004.tif]

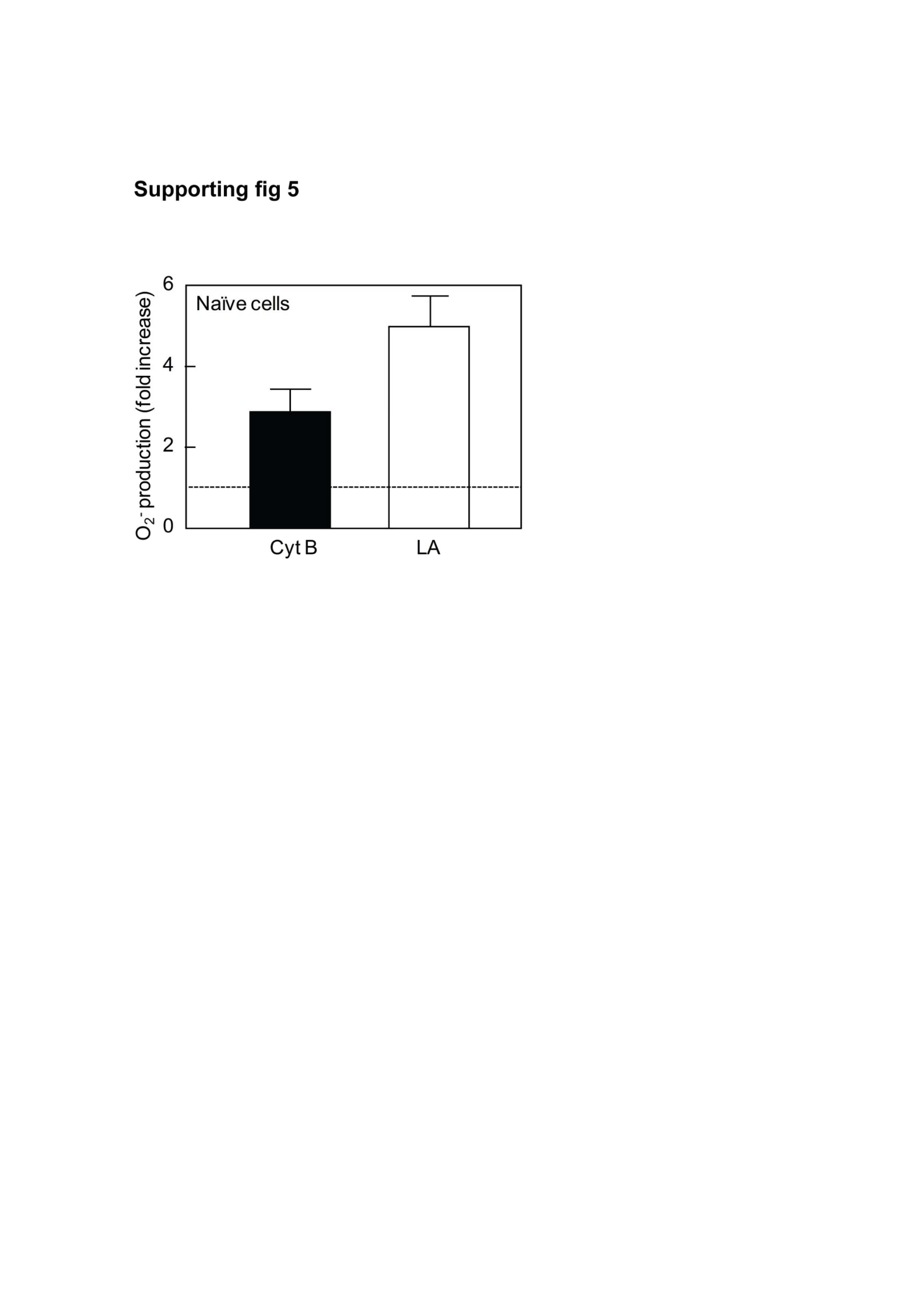

Supplement: Figure S5 — The PAF-induced neutrophil response is primed by inhibitors of actin polymerization. Naïve human neutrophils were incubated at 37°C for 5 min with either Cytochalasin B (Cyt B, 5 µg/ml; grey bars) or latrunculin A (LA, 50 ng/ml; white bars). Control cells were incubated at the same conditions but in the absence of actin polymerization inhibitor. The cells were then activated with PAF (100 nM) and the release of superoxide was recorded continuously. Data are expressed as fold increase of peak values in treated cells as compared to non-treated controls (mean ± SEM; n = 3). The dashed line denotes the value expected in the absence of effect. (TIF) [file pone.0060169.s005.tif]

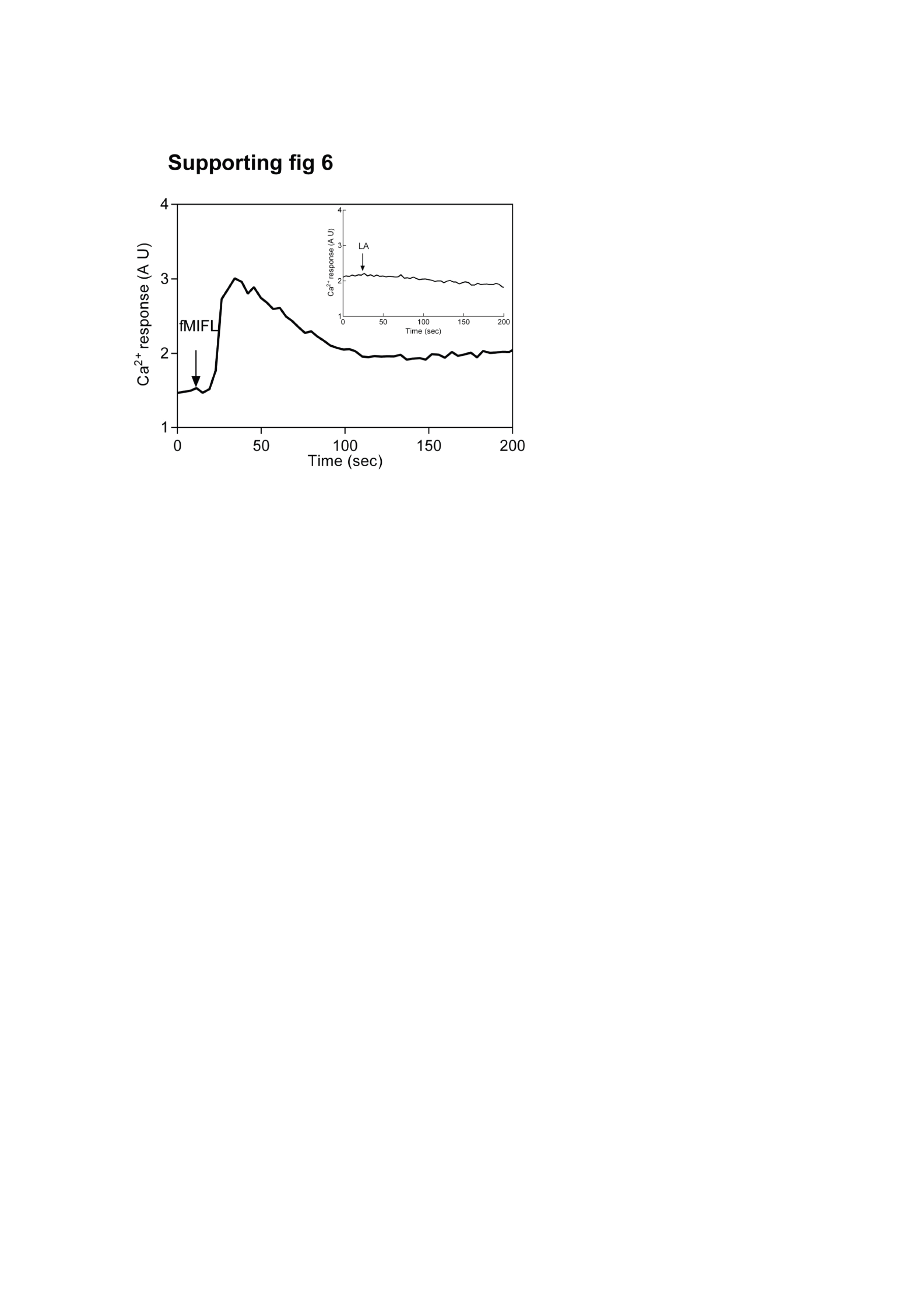

Supplement: Figure S6 — Latrunculin A induces no increase in intracellular Ca2+ in FPR1des neutrophils. Intracellular Ca2+ canges was determined in Fura-2 loaded naïve and FPR1des (0.1 nM fMIFL) neutrophils. Naïve neutrophils were activated by fMIFL (1 nM; solid line), and FPR1des neutrophils were reactivated by latrunculin A (100 ng/ml; inset). The changes in fluorescence were followed using dual excitation at 340 nm and 380 nm, and an emission wavelength of 510 nm. Representative experiments are shown. Abscissa, time of study (min); Ordinate, relative change in [Ca2+]i. (TIF) [file pone.0060169.s006.tif]
